# ^18^F-FDG PET/CT-based radiomics nomogram for the preoperative prediction of lymph node metastasis in gastric cancer

**DOI:** 10.3389/fonc.2022.911168

**Published:** 2022-08-08

**Authors:** Xiu-qing Xue, Wen-Ji Yu, Xun Shi, Xiao-Liang Shao, Yue-Tao Wang

**Affiliations:** ^1^ Department of Nuclear Medicine, The First People’s Hospital of Yancheng, Yancheng, China; ^2^ The Yancheng Clinical College of Xuzhou Medical University, Yancheng, China; ^3^ Department of Nuclear Medicine, The Third Affiliated Hospital of Soochow University, Changzhou, China; ^4^ Institute of Clinical Translation of Nuclear Medicine and Molecular Imaging, Soochow University, Changzhou, China

**Keywords:** gastric cancer, positron emission tomography - computed tomography (PET-CT), radiomics, nomogram, lymph node metastasis (LNM)

## Abstract

**Objective:**

Lymph node metastasis (LNM) is not only one of the important factors affecting the prognosis of gastric cancer but also an important basis for treatment decisions. The purpose of this study was to investigate the value of the radiomics nomogram based on preoperative ^18^F-deoxyglucose (FDG) PET/CT primary lesions and clinical risk factors for predicting LNM in gastric cancer (GC).

**Methods:**

We retrospectively analyzed radiomics features of preoperative ^18^F-FDG PET/CT images in 224 gastric cancer patients from two centers. The prediction model was developed in the training cohort (n = 134) and validated in the internal (n = 59) and external validation cohorts (n = 31). The least absolute shrinkage and selection operator (LASSO) regression was used to select features and build radiomics signatures. The radiomics feature score (Rad-score) was calculated and established a radiomics signature. Multivariate logistic regression analysis was used to screen independent risk factors for LNM. The minimum Akaike’s information criterion (AIC) was used to select the optimal model parameters to construct a radiomics nomogram. The performance of the nomogram was assessed with calibration, discrimination, and clinical usefulness.

**Results:**

There was no significant difference between the internal verification and external verification of the clinical data of patients (all *p* > 0.05). The areas under the curve (AUCs) (95% CI) for predicting LNM based on the ^18^F-FDG PET/CT radiomics signature in the training cohort, internal validation cohort, and external validation cohort were 0.792 (95% CI: 0.712–0.870), 0.803 (95% CI: 0.681–0.924), and 0.762 (95% CI: 0.579–0.945), respectively. Multivariate logistic regression showed that carbohydrate antigen (CA) 19-9 [OR (95% CI): 10.180 (1.267–81.831)], PET/CT diagnosis of LNM [OR (95% CI): 6.370 (2.256–17.984)], PET/CT Rad-score [OR (95% CI): 16.536 (5.506–49.660)] were independent influencing factors of LNM (all *p* < 0.05), and a radiomics nomogram was established based on those factors. The AUCs (95% CI) for predicting LNM were 0.861 (95% CI: 0.799–0.924), 0.889 (95% CI: 0.800–0.976), and 0.897 (95% CI: 0.683–0.948) in the training cohort, the internal validation cohort, and the external validation cohort, respectively. Decision curve analysis (DCA) indicated that the ^18^F-FDG PET/CT-based radiomics nomogram has good clinical utility.

**Conclusions:**

Radiomics nomogram based on the primary tumor of ^18^F-FDG PET/CT could facilitate the preoperative individualized prediction of LNM, which is helpful for risk stratification in GC patients.

## Main findings

- The radiomics nomogram based on preoperative ^18^F-FDG PET/CT primary lesions and clinical risk factors showed a favorable performance for predicting LNM in GC.
- PET/CT Rad-score outperformed conventional PET/CT diagnosis of LNM in GC.
- The radiomics nomogram facilitates the preoperative individualized prediction of LNM in GC.

## Introduction

In China, gastric cancer (GC) is the most common malignant tumor of the digestive system (1). Lymph node metastasis (LNM) is the most common route of GC metastasis, which not only affects the prognosis of GC patients but also affects the formulation of personalized treatment plans (1, 2). Accurately evaluating lymph node status before an operation is significant for making pretreatment decisions, dissecting lymph nodes intraoperatively, and selecting the surgical methods and the neoadjuvant chemotherapy (3–7). The existing imaging methods for diagnosing LNM of GC include computed tomography (CT), endoscopic ultrasonography (EUS), and magnetic resonance imaging (MRI). However, the above imaging methods diagnosing LNM of GC are mainly based on the information of lymph nodes, such as the diameter and enhancement of lymph nodes, and their diagnostic accuracy is still not satisfactory (8). Since LNM is a complex biological process, it can also be affected by characteristics of primary tumor cells, such as proliferation rate, invasiveness, and chemotaxis (9, 10). With the development of molecular imaging technology, ^18^F-fluoro-2-deoxy-glucose positron emission tomography/computed tomography (^18^F-FDG PET/CT) has been clinically applied in the staging, restaging, curative effect evaluation, and prognosis evaluation in GC, which is advantageous in evaluating tumor metabolism (11–13). However, the conventional ^18^F-FDG PET/CT diagnosis has the drawback of low sensitivity (<50%) for detecting LNM in GC, due to low spatial resolution and some lesions without obvious ^18^F-FDG uptake (14–16). Understanding these heterogeneities associated with tumor metabolic properties requires further analysis of the images. Radiomics analysis refers to the quantitative extraction of high-dimensional data from medical digital images to realize the non-invasive analysis of tumor heterogeneity (17), which gradually expanded to PET/CT imaging applications, and it is necessary to use radiomics to improve the accuracy of predicting LNM. Most of the radiomics research was based on CT or MR (18–20); there were few ^18^F-FDG PET/CT-based studies that would enable excellent prediction of LNM in GC. It is well known that the metabolic information obtained from ^18^F-FDG PET/CT in the primary tumors may reflect the biological aggressiveness and proliferative activity of the tumor, which is always related to tumor LNM (21). Furthermore, radiomics analysis based on ^18^F-FDG PET/CT is advantageous in that it can merge the metabolic information from ^18^F-FDG PET with the anatomical information from CT, so radiomics deeply explore the potential connection between medical images and LNM and may be more helpful to predict LNM than CT and MRI alone. Therefore, it is necessary to use radiomics based on ^18^F-FDG PET/CT from the primary tumor to improve the accuracy of predicting LNM.

The purpose of this study was to establish and estimate the value of the radiomics nomogram based on ^18^F-FDG PET/CT imaging for the individual preoperative prediction of LNM in GC.

## Methods

### Patient selection

**Figure 1:**
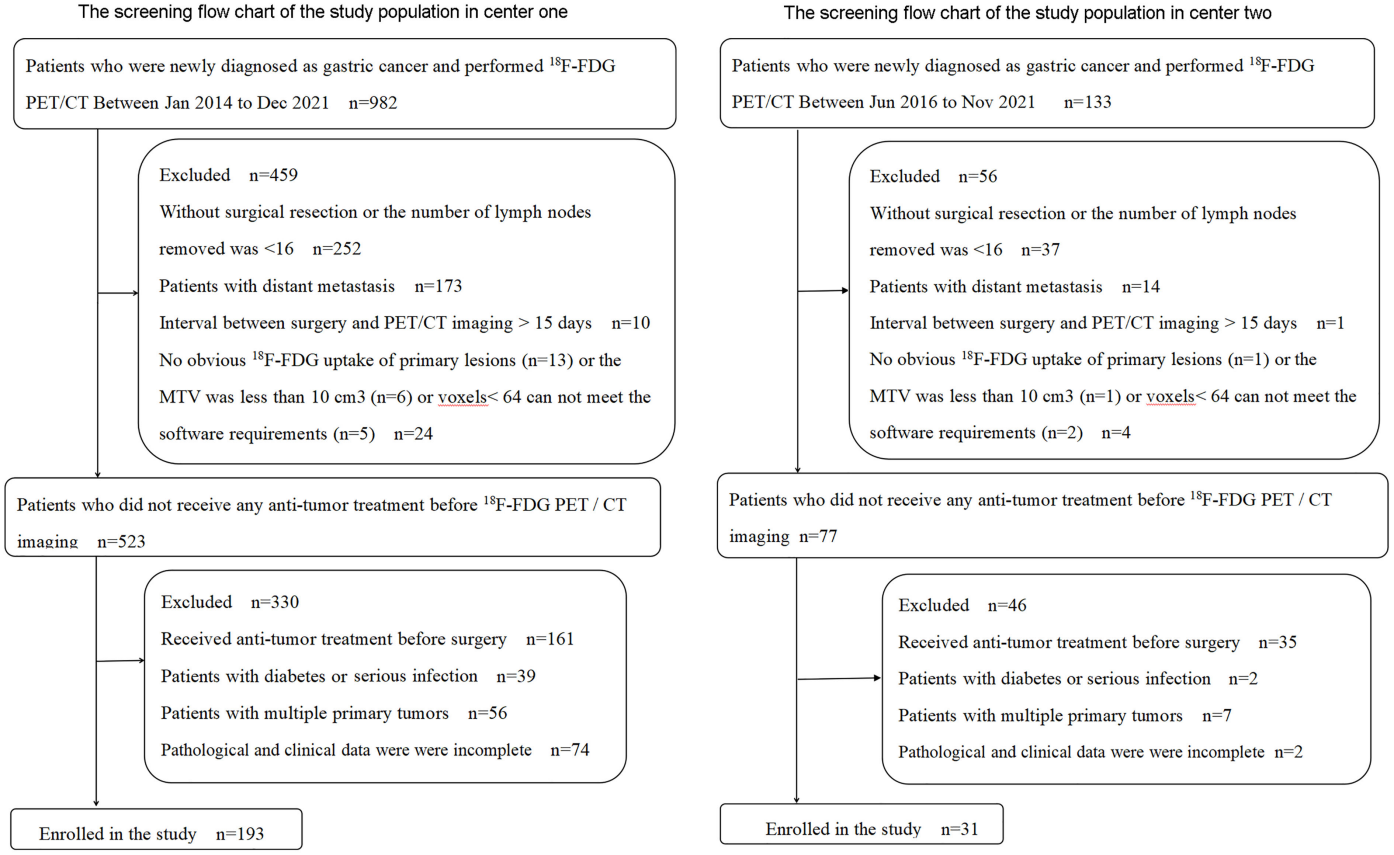
Flowchart of the study population.

A total of 224 GC patients were enrolled in the training cohort and internal validation cohort (n = 193) from the Third Affiliated Hospital of Soochow University (study center 1) and external validation cohort (n = 31) from the First People’s Hospital of Yancheng (study center 2) between January 2014 and December 2021. Inclusion criteria were 1) patients with a pathologically confirmed GC, 2) an interval of <15 days between surgery and PET/CT imaging, 3) PET/CT images met the analysis requirements, and 4) complete postoperative pathological and clinical data. Exclusion criteria were 1) subjects had undergone any anti-tumor treatment before surgery, 2) the number of lymph nodes removed was <16 by D2 lymphadenectomy, 3) subjects with multiple primary tumors, 4) subjects with a serious infection or diabetes, and 5) lesions were without obvious ^18^F-FDG uptake (study center 1 has 13 patients, and study center 2 has 1 patient) or the metabolic tumor volume (MTV) was less than 10 cm^3^ or voxels < 64 cannot meet the software requirements. The screening flowchart of the study population is shown in **Figure 1**.

Basic information, clinical data, and the postoperative pathology of all the subjects were collected, including gender, age, body mass index (BMI), primary tumor location, the state of lymph nodes, C-reactive protein (CRP), and preoperative tumor biomarkers including carbohydrate antigen (CA) 19-9 and carcinoembryonic antigen (CEA). The post-operative staging was evaluated based on the TNM 8th edition (22). The retrospective analysis was approved by the ethics committee of the Third Affiliated Hospital of Soochow University (study center 1) and the First People’s Hospital of Yancheng (study center 2). The requirement for informed consent was waived.

### PET/CT imaging procedure

All patients underwent PET/CT after fasting for at least 6 h and were checked for blood glucose levels ≤ 10.0 mmol/L. Imaging acquisition began 1 h after ^18^F-FDG (radiochemical purity > 95%) was injected at a dose of 4.44 MBq/kg. The ^18^F-FDG of study center 1 and study center 2 was provided by JYAMS PET Research and Development Ltd. (Nanjing, China) and Shanghai Atom Kexing Pharmaceuticals Co., Ltd. (Shanghai, China), respectively. Images were obtained from the skull base to upper thighs using the same PET/CT equipment of the models (Biograph mCT 64 PET/CT system: Siemens Medical Solutions, Ann Arbor, MI, USA) in two study centers. All the subjects were instructed to drink at least 500 ml of water prior to PET/CT scanning. Low-dose CT scanning (study center 1: 35 mA/120 kV, 5-mm thickness; study center 2: automatic regulating current/120 kV, 5-mm thickness) was used for attenuation correction. PET images were obtained with an acquisition time of 2 min per bed position in a three-dimensional (3D) model and reconstructed by the ordered subset expectation maximization (OSEM).

### The tumor site of primary tumors

In accordance with the previous studies (19, 21, 23), the stomach is divided into three parts (upper, middle, and lower) based on the tripartite connection of the greater curvature and the lesser curvature at the position of the coronal.

### Imaging analysis and radiomics feature extraction

The PET/CT images in the two study centers were transferred to the LIFEx software in DICOM format. For PET/CT imaging, radiomics features were extracted by using the LIFEx software (version 6.0, http://www.lifexsoft.org ) from the volume of interest (VOI) of the primary tumor lesion (24). For PET images, we adopted the threshold of 40% of the SUVmax to semiautomatically define the VOI for the primary lesion. The segmentation correction was performed by two nuclear medicine doctors (Y.WJ outlined the VOI, and X.XQ checked it). If there is any dispute, they would discuss it with the third nuclear medicine doctor and solve it through negotiation. When the respiratory movement caused the mismatch between CT and PET images, the VOI of the primary lesion on the CT image was manually adjusted. Intensity discretization is for PET images and CT images (PET images, the continuous scale decreased to 64 bins with absolute scale bounds between 0 and 30; CT images, gray levels of 400 bins with absolute scale bounds between −10,000 and 3,000 HU). LIFEx software automatically extracted 136 textural features (PET, 71 features; CT, 65 features) from the VOI of the PET/CT image (**Table 1**). When using texture features of PET images to quantify the heterogeneity, it could be affected by the tumor volume effect, particularly in the focus zone, which is less than 10 cm^3^ (25); thus, we only conducted analyses for MTV greater than 10 cm^3^. The intra- and interobserver reproducibility and consistency of the PET/CT texture feature extraction were evaluated by intra- and interclass correlation coefficients (ICCs). ICC > 0.75 manifested a consistency of extraction properties.

**Table 1:** PET/CT radiomics feature extraction of primary gastric cancer.

Index	Parameter
First-order features	
Conventional indices	SUVmin, SUVmean, SUVmax, SUVpeak, SUVStd, SUVQ1, SUVQ2, SUVQ3, SUVSkewness, SUVKurtosis, SUVExcessKurtosis, SUVpeakSphere0.5mL, SUVpeakSphere1mL, TLG(mL)HUmin, HUmean, HUmax, HUpeak, HUStd, HUQ1, HUQ2, HUQ3, HUSkewness, HUKurtosis, HUExcessKurtosis
Discretized indices	SUVmin, SUVmean, SUVmax, SUVpeak, SUVStd, SUVQ1, SUVQ2, SUVQ3, SUVSkewness, SUVKurtosis, SUVExcessKurtosis, SUVpeakSphere0.5mL, SUVpeakSphere1mL, TLG(mL), HUmin, HUmean, HUmax, HUpeak, HUStd, HUQ1, HUQ2, HUQ3, HUSkewness, HUKurtosis, HUExcessKurtosis, HISTO_Skewness, HISTO_Kurtosis, HISTO_ExcessKurtosis, HISTO_Entropy_log10, HISTO_Entropy_log2, HISTO_Energy
Shape-derived parameters	SHAPE_Volume(mL), SHAPE_Volume(vx), SHAPE_Sphericity, SHAPE_Surface(mm2), SHAPE_Compacity
Texture features	
GLCM	Homogeneity, energy, contrast, correlation, Entropy_log10, Entropy_log2, Dissimilarity
GLRLM	SRE, LRE, LGRE, HGRE, SRLGE, SRHGE, LRLGE, LRHGE, GLNU, RLNU, RP
NGLDM	Coarseness, contrast, busyness
GLZLM	SZE, LZE, LGZE, HGZE, SZLGE, SZHGE, LZLGE, LZHGE, GLNU, ZLNU, ZP

TLG, total lesion glycolysis; GLCM, gray-level co-occurrence matrix; GLRLM, gray-level run length matrix; SRE, short-run emphasis; LRE, long-run emphasis; LGRE, low gray-level run emphasis; HGRE, high gray-level run emphasis; SRLGE, short run low gray-level emphasis; SRHGE, short run high gray-level emphasis; LRLGE, long-run low gray-level emphasis; LRHGE, long-run high gray-level emphasis; GLNU, gray-level non-uniformity; RLNU, run length non-uniformity; RP, run percentage; NGLDM, neighborhood gray-level difference matrix; GLZLM, gray-level zone-length matrix; SZE, short-zone emphasis; LZE, long-zone emphasis; LGZE, low gray-level zone emphasis; HGZE, high gray-level zone emphasis; SZLGE, short-zone low gray-level emphasis; SZHGE, short-zone high gray-level emphasis; LZLGE, long-zone low gray-level emphasis; LZHGE, long-zone high gray-level emphasis; GLNU, gray-level non-uniformity; ZLNU, zone-length non-uniformity; ZP, zone percentage.

### Radiomics feature selection and model construction

All the extracted radiomics features were standardized by Z-score, and the data were converted into a new score with an average value of 0 and a standard deviation of 1. Due to the relatively small sample size and the relatively large radiomics features, the Mann–Whitney U test was primarily applied to filter some features with significant differences between the LNM group and the no LNM (NLNM) group (*p* < 0.10). Then, the least absolute shrinkage and selection operator (LASSO) regression (26) was used to screen out the effective features, and the radiomics feature score (Rad-score) was calculated and established a radiomics prediction model. PET-score only included the PET radiomics feature, and PET/CT-score simultaneously contained the PET and CT radiomics feature. Multivariate logistic regression analysis was used to screen independent risk factors for LNM, and the minimum Akaike’s information criterion (AIC) was used to select the optimal model parameters to construct a radiomics nomogram.

### The diagnosis of lymph node metastasis by conventional ^18^F-FDG PET/CT

The diagnosis of LNM in GC by conventional ^18^F-FDG PET/CT was defined as follows: the diameter of lymph nodes ≥ 10 mm on CT images or the ^18^F-FDG uptake of lymph nodes on PET images was similar to or higher than that of the liver (27).

### Histopathological diagnostic criteria for lymph node metastasis

Conventional paraffin-embedded specimens were sectionalized at 4–5 μm continuously and stained with hematoxylin and eosin (H&E). The diagnostic criteria for LNM are the presence of carcinoma in the lymph node capsule. Each slice was interpreted separately by two pathologists. In case of disagreement, the third pathologist shall be invited to interpret it again. Finally, three pathologists will reach a consensus through consultation.

### Statistical analysis

Continuous variables were indicated as mean±standard deviation(SD) or M (p25-p75), and categorical variables were indicated as frequency (%). We compared two groups using the Mann–Whitney U tests or independent t-tests for continuous variables. Pearson’s chi-square test or Fisher’s exact test was used for categorical variables. Univariate and multivariable logistic regression analyses were conducted to identify the independent risk factors for LNM. The collinearity between significant independent variables was evaluated using correlation coefficients β and variance inflation factors (VIFs), and only variables with correlation coefficients β < 0.25 or VIF < 5 were included. A violin plot was used to show the distribution and probability density of PET/CT-scores. Receiver operating characteristic (ROC) and decision curves were used to evaluate and validate the performance of nomograms for predicting LNM in the training cohort, internal validation cohort, and external validation cohort. Internal validation of the model and calculation of the 95% confidence interval (CI) of the area under the curve (AUC) were performed using the bootstrap resampling method (times = 500), which was recommended by the Tripod Reporting Specification (28). All the data analyses were conducted with R3.4.3 (http://www.R-project.org; software packages: glmnet, pROC, rms, dca. R). A two-sided *p* < 0.05 was considered statistically significant.

## Results

### Basic characteristics of the enrolled patients

Cases from study center 1 were randomly divided into a training cohort (n = 134) and an internal validation cohort (n = 59) in a 7:3 ratio. Baseline characteristics of the patients in the training cohort (n = 134), internal (n = 59), and external validation cohort (n = 31) are listed in **Tables 2**, **3**. The clinical characteristics of patients were similar among the three cohorts (all *p* > 0.05): 66.4% (89/134), 66.1% (39/59), and 67.7% (21/31) patients have LNM in the training cohort, internal validation cohort, and external validation cohort, respectively. According to the postoperative pathological results, 649 of 2,989 LNs, 318 of 1,329 LNs, and 101 of 578 LNs were found to be positive for metastasis in the training cohort, internal validation cohort, and external validation cohort, respectively. The levels of CA19-9 and CEA included in the study were used as categorical variables. In the training cohort, the proportion of CA19-9 > 37 and PET/CT diagnosis positive in the LNM group were significantly higher than that of the NLNM group (17.98% *vs*. 2.22%, *p* = 0.01; 43.82% *vs*. 11.11%, *p* < 0.001). Other clinical factors, including age, gender, BMI, CEA, CRP, location of primary focus, and pathological type showed no significant differences between the two groups (*p* = 0.084–0.978). In the internal validation cohort, the proportion of CA19-9 > 37 in the LNM group was significantly higher than that in the NLNM group (20.51% *vs*. 0.00%, *p* = 0.029), and the other clinical characteristics were not significantly different between the two groups (*p* = 0.093–0.885). In the external validation cohort, there were no statistically significant differences between the LNM group and NLNM group in all clinical characteristics (*p* = 0.071–0.095).

**Table 2:** Characteristics of the study patients in two centers.

Characteristics	Training cohort	Internal validation cohort	External validation cohort	*p*
Cases (n)	134	59	31	
Age, mean ± SD, years	65.5 ± 10.6	64.6 ± 12.3	63.6 ± 13.4	0.699
BMI, mean ± SD, (kg/m2)	22.7 ± 3.0	22.9 ± 2.9	23.8 ± 2.6	0.171
Gender, n (%)				0.940
Male	93 (69.40%)	42 (71.19%)	21 (67.74%)	
Female	41 (30.60%)	17 (28.81%)	10 (32.26%)	
CEA, n (%)				0.324
≤5	108 (80.60%)	42 (71.19%)	25 (80.65%)	
>5	26 (19.40%)	17 (28.81%)	6 (19.35%)	
CA19-9, n (%)				0.171
≤37	117 (87.31%)	51 (86.44%)	23 (74.19%)	
>37	17 (12.69%)	8 (13.56%)	8 (25.81%)	
CRP, n (%)				0.865
≤10	29 (21.64%)	14 (23.73%)	8 (25.81%)	
>10	105 (78.36%)	45 (76.27%)	23 (74.19%)	
Conventional PET/CT diagnosis of LNM				0.783
Negative	90 (67.16%)	42 (71.19%)	20 (64.52%)	
Positive	44 (32.84%)	17 (28.81%)	11 (35.48%)	
Pathological diagnosis of LNM				0.987
Negative	45 (33.58%)	20 (33.90%)	10 (32.26%)	
Positive	89 (66.42%)	39 (66.10%)	21 (67.74%)	
Tumor site, n (%)				0.439
Upper	40 (29.85%)	22 (37.29%)	14 (45.16%)	
Middle	39 (29.10%)	15 (25.42%)	9 (29.03%)	
Lower	55 (41.05%)	22 (37.29%)	8 (25.81%)	
Pathological types, n (%)				0.462
Ade	114 (85.08%)	45 (76.28%)	27 (87.09%)	
Ade + sig	12 (8.96%)	6 (10.17%)	1 (3.23%)	
Ade + mus	3 (2.23%)	1 (1.69%)	0 (0.00%)	
Ade + sig + mus	1 (0.75%)	1 (1.69%)	0 (0.00%)	
Others	4 (2.98%)	6 (10.17%)	3 (9.68%)	
TNM stage				0.155
Stage I	35 (26.12%)	13 (22.03%)	4 (12.90%)	
Stage II	28 (20.89%)	9 (15.25%)	11 (35.48%)	
Stage III	71 (52.99%)	37 (62.71%)	16 (51.61%)	

BMI, body mass index; CEA, carcinoembryonic antigen; CA19-9, carbohydrate antigen 19-9; CRP, C-reactive protein; Ade, adenocarcinoma; Mus, mucous adenocarcinoma; sig, signet-ring cell carcinoma; Others, pure mucous adenocarcinoma or pure signet-ring cell carcinoma.

**Table 3:** Characteristics of patients in training cohort, internal validation cohort, and external validation cohort.

Characteristics	Training set (n = 134)		*p*-Value	Internal validation set (n = 59)		*p*-Value	External validation set (n = 31)		*p*-Value
	LNM, n = 89	NLNM, n = 45		LNM, n = 39	NLNM, n = 20		LNM, n = 21	NLNM, n = 10	
Age, mean ± SD, years	65.8 ± 10.1	64.9 ± 11.5	0.649	66.1 ± 11.0	61.7 ± 14.41	0.199	64.9 ± 13.4	61.0 ± 13.18	0.459
Gender, n (%)			0.200			0.885			0.853
Male	65 (73.03%)	28 (62.22%)		28 (71.79%)	14 (70.00%)		14 (66.67%)	7 (70.00%)	
Female	24 (26.97%)	17 (37.78%)		11 (28.21%)	6 (30.00%)		7 (33.33%)	3 (30.00%)	
BMI, mean ± SD (kg/m^2^)	22.5 ± 2.9	22.9 ± 3.1	0.412	22.8 ± 2.7	23.2 ± 3.2	0.597	23.5 ± 2.4	24.3 ± 3.1	0.457
CEA, n (%)			0.084			0.093			0.950
≤5	68 (76.41%)	40 (88.89%)		25 (64.10%)	17 (85.00%)		17 (80.95%)	8 (80.00%)	
>5	21 (23.59%)	5 (11.11%)		14 (35.90%)	3 (15.00%)		4 (19.05%)	2 (20.00%)	
CA-199, n (%)			0.010			0.029			0.713
≤37	73 (82.02%)	44 (97.78%)		31 (79.49%)	20 (100.00%)		16 (76.19%)	7 (70.00%)	
>37	16 (17.98%)	1 (2.22%)		8 (20.51%)	0 (0.00%)		5 (17.98%)	3 (23.81%)	
CRP, n (%)			0.575			0.869			0.713
≤10	18 (20.23%)	11 (24.44%)		9 (23.08%)	5 (25.00%)		5 (23.81%)	3 (30.00%)	
>10	71 (79.77%)	34 (75.56%)		30 (76.92%)	15 (75.00%)		16 (76.19%)	7 (70.00%)	
Tumor site, n (%)			0.978			0.633			0.071
Upper	27 (30.34%)	13 (28.89%)		16 (41.03%)	6 (30.00%)		10 (47.62%)	4 (40.00%)	
Middle	26 (29.21%)	13 (28.89%)		10 (25.64%)	5 (25.00%)		8 (38.10%)	1 (10.00%)	
Lower	36 (40.45%)	19 (42.22%)		13 (33.33%)	9 (45.00%)		3 (14.28%)	5 (50.00%)	
Pathological types, n (%)			0.388			0.303			0.782
Ade	74 (83.15%)	40 (88.89%)		27 (69.23%)	18 (90.00%)		18 (85.72%)	9 (90.00%)	
Ade + mus	3 (3.37%)	0 (0.00%)		1 (2.56%)	0 (0.00%)		0 (0.00%)	0 (0.00%)	
Ade + sig	9 (10.11%)	3 (6.67%)		4 (10.26%)	2 (10.00%)		1 (4.76%)	0 (0.00%)	
Ade + sig + mus	0 (0.00%)	1 (2.22%)		1 (2.56%)	0 (0.00%)		0 (0.00%)	0 (0.00%)	
Others	3 (3.37%)	1 (2.22%)		6 (15.39%)	0 (0.00%)		2 (9.52%)	1 (10.00%)	
Conventional PET/CT diagnosis of LNM			<0.001			0.093			0.214
Positive	39 (43.82%)	5 (11.11%)		14 (35.90%)	3 (15.00%)		9 (42.86%)	2 (20.00%)	
Negative	50 (56.18%)	40 (88.89%)		25 (64.10%)	17 (85.00%)		12 (57.14%)	8 (80.00%)	

LNM, lymph node metastasis; NLNM, non-lymph node metastasis; BMI, body mass index; CEA, carcinoembryonic antigen; CA19-9, carbohydrate antigen 19-9; CRP, C-reactive protein; Ade, adenocarcinoma; Mus, mucous adenocarcinoma; sig, signet-ring cell carcinoma; Others, pure mucous adenocarcinoma or pure signet-ring cell carcinoma.

### Feature extraction and selection

**Figure 2:**
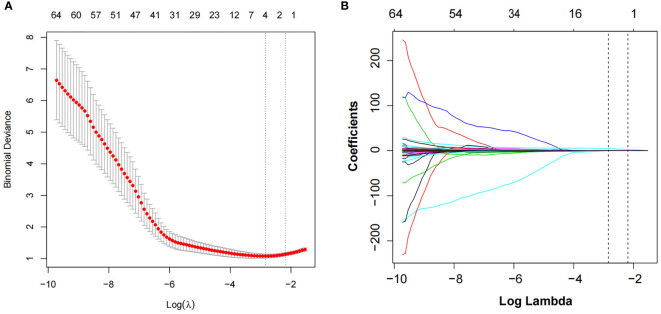
LASSO algorithm and 10-fold cross-validation to select the optimal texture features. **(A)** The tuning parameter (λ) in the LASSO model was selected using 10-fold cross-validation. The function of log(λ) is plotted by binomial deviances from the LASSO regression cross-validation. The black vertical line is plotted at the best values of λ for which the model provides the best matching of the data. λ = 0.1122 with log (λ) = −2.1872 was chosen as the optimal value. **(B)** LASSO coefficient profiles of the 64 radiomics features. The vertical line is the value selected using 10-fold cross-validation in panel A, where optimal λ resulted in two non-zero coefficients. LASSO, least absolute shrinkage and selection operator.

The intra-observer ICCs of the texture features were from 0.911 to 0.982, whereas the interobserver ICCs were from 0.961 to 0.990. Of the texture features, 136 features were reduced to 64 potential predictors on the basis of 193 patients in the training cohort. Then λ = 0.1122 with log (λ) = −2.1872 was chosen as the optimal value, and finally, the optimal λ resulted in two non-zero coefficients (**Figure 2**). The two non-zero coefficients included were GLZLM_SZE (PET feature) and CONVENTIONAL_HUQ3 (CT feature). The Rad-score of each patient was calculated by the following calculation formula:

PET/CT-score = 2.74891 * GLZLM_SZE + 0.01145 * CONVENTIONAL_HUQ3

### Comparison of PET-score and PET/CT-score in training cohort and verification cohort (internal and external)

**Figure 3:**
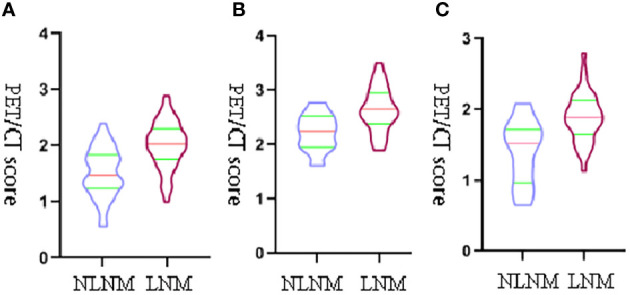
The violin figure of the PET/CT-score. The transverse axis represents the status of lymph nodes, and the vertical y-axis represents PET/CT-score. **(A)** The violin figure of the PET/CT-score between the LNM group and NLNM group in training set. **(B)** The violin figure of the PET/CT-score between the LNM group and NLNM group in internal validation set. **(C)** The violin figure of the PET/CT-score between the LNM group and NLNM group in external validation set.

The comparison of the PET-score and PET/CT-score between the LNM group and NLNM group in the training cohort, internal cohort, and external validation cohort is shown in **Table 4**. The results showed that PET-score and PET/CT-score in the LNM group were much higher than those in the NLNM group (*p* < 0.05 in the three cohorts). The PET-scores of the LNM group and NLNM group were (1.634 (1.311–1.875) *vs*. 1.173 (0.855–1.505), *p* < 0.001), (1.470 (1.264–1.691) *vs*. 1.092 (0.632–1.320), *p* < 0.001), and (1.486 (1.254–1.726) *vs*. 1.079 (0.806–1.297), *p* = 0.009) in the training cohort, internal validation cohort, and external validation cohort, respectively. The PET/CT-scores of the LNM group and NLNM group were [2.033 (1.767–2.287) *vs*. 1.474 (1.252–1.817), *p* < 0.001), (1.890 (1.693–2.093) *vs*. 1.518 (1.071–1.713), *p* < 0.001), and (1.852 (1.565–2.235) *vs*. 1.462 (1.194–1.736), *p* = 0.011] in the training cohort, internal validation cohort, and external validation cohort, respectively. The violin figure of the Rad-score is displayed in **Figure 3**.

**Table 4:** Comparison between the PET-score and PET/CT-score in training set and verification set.

	NLNM group	LNM group	*p*-Value
Training set			
PET-score	1.173 (0.855–1.505)	1.634 (1.311–1.875)	<0.001
PET/CT-score	1.474 (1.252–1.817)	2.033 (1.767–2.287)	<0.001
Internal validation set			
PET-score	1.092 (0.632–1.320)	1.470 (1.264–1.691)	<0.001
PET/CT-score	1.518 (1.071–1.713)	1.890 (1.693–2.093)	<0.001
Subjects in center 1			
PET-score	1.156 (0.811–1.405)	1.549 (1.301–1.799)	<0.001
PET/CT-score	1.517 (1.198–1.797)	1.973 (1.735–2.213)	<0.001
External validation set			
PET-score	1.079 (0.806–1.297)	1.486 (1.254–1.726)	0.009
PET/CT-score	1.462 (1.194–1.736)	1.852 (1.565–2.235)	0.011

PET-score and PET/CT-score were expressed as [median (p25–p75)].

LNM, lymph node metastasis; NLNM, no lymph node metastasis.

### Construction and verification of radiomics nomogram

**Figure 4:**
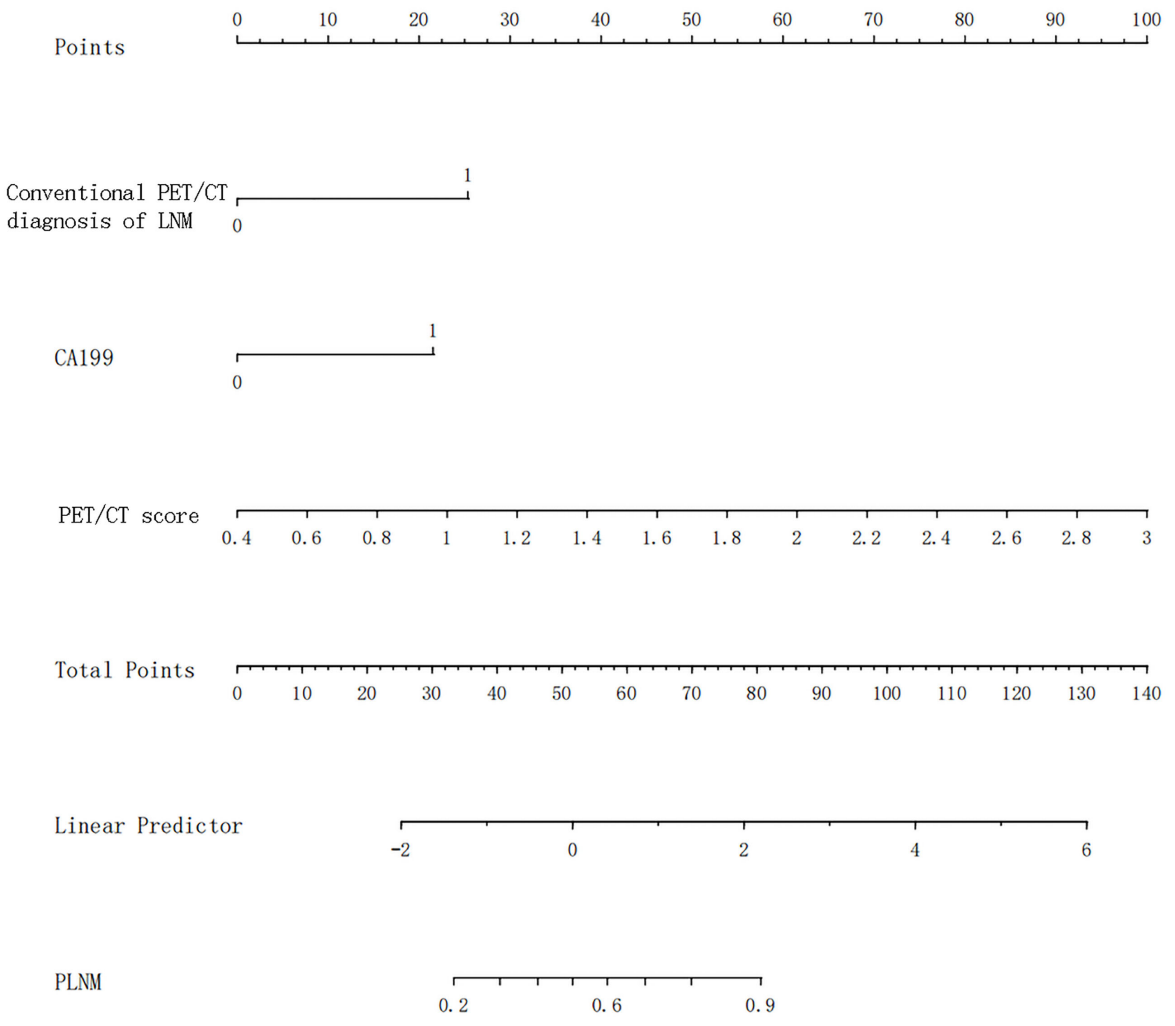
The nomogram for the prediction of LNM. LNM, lymph node metastasis.

Univariate and multivariate logistic regression results are shown in **Table 5**. Multivariate logistic regression showed that CA19-9 [OR (95% CI): 10.180 (1.267–81.831)], PET/CT diagnosis of LNM [OR (95% CI): 6.370 (2.256–17.984)], and PET/CT score [OR (95% CI): 16.536 (5.506–49.660)] were independent influencing factors of LNM in GC (all *p* < 0.05). Then, we constructed a radiomics nomogram, integrating CA19-9, PET/CT diagnosis of LNM, and PET/CT Rad-score, according to the AIC in the training cohort (**Figure 4**). The nomogram formula is as follows:

**Table 5:** Univariate and multivariate logistic regression analyses for lymph node metastasis.

	Univariate logistic regression		Multivariate logistic regression	
Variables	OR (95% CI)	*p*-Value	OR (95% CI)	*p*-Value
Age	1.008 (0.974, 1.043)	0.6459	1.008 (0.974, 1.043)	0.6572
BMI	0.951 (0.844, 1.072)	0.4094	0.948 (0.840, 1.071)	0.3943
Gender (female)	0.608 (0.284, 1.304)	0.2015	0.593 (0.275, 1.278)	0.1820
CEA	2.471 (0.864, 7.064)	0.0915	2.702 (0.917, 7.967)	0.0715
CA19-9	9.644 (1.236, 75.261)	0.0306	10.180 (1.267, 81.831)	0.0291
CRP	1.276 (0.543, 2.998)	0.5758	1.245 (0.523, 2.963)	0.6199
Conventional PET/CT diagnosis of LNM	6.240 (2.251, 17.298)	0.0004	6.370 (2.256, 17.984)	0.0005
PET/CT-score	14.336 (5.059, 40.626)	<0.0001	16.536 (5.506, 49.660)	<0.0001

BMI, body mass index; CEA, carcinoembryonic antigen; CA19-9, carbohydrate antigen 19-9; CRP, C-reactive protein; LNM, lymph node metastasis.

Logit (LNM) = −5.08113 + 1.92293 * (traditional PET/CT diagnosis LNM = positive) + 1.63248 * (CA19-9 > 37) + 2.91691 * PET/CT-score

**Figure 5:**
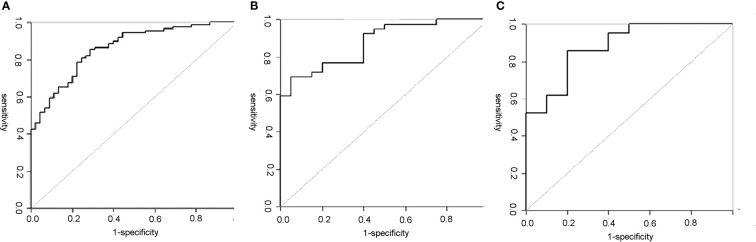
ROC curves of the PET/CT radiomics nomogram in each cohort. **(A)** The ROC curve of the PET/CT radiomics nomogram for predicting LNM in training set; the AUC was 0.861. **(B)** The ROC curve of the PET/CT radiomics nomogram for predicting LNM in internal validation set; the AUC was 0.889. **(C)** The ROC curve of the PET/CT radiomics nomogram for predicting LNM in external validation set; the AUC was 0.897. ROC, receiver operating characteristic; LNM, lymph node metastasis; AUC, area under the curve.

**Figure 6:**
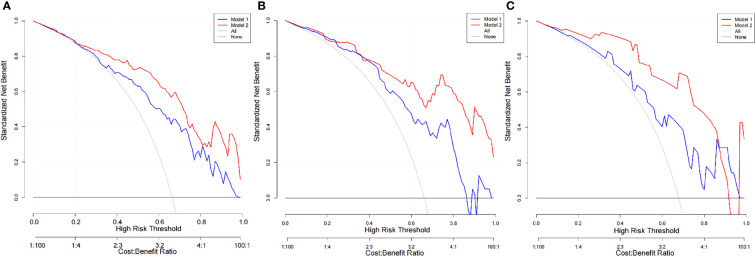
DCA of the PET/CT-score and PET/CT radiomics nomogram in each cohort. **(A)** DCA of the PET/CT-score and PET/CT radiomics nomogram in training set (Model 1, PET/CT-score; Model 2, PET/CT radiomics nomogram). **(B)** DCA of the PET/CT-score and PET/CT radiomics nomogram in internal validation set (Model 1, PET/CT-score; Model 2, PET/CT radiomics nomogram). **(C)** DCA of the PET/CT-score and PET/CT radiomics nomogram in external validation set (Model 1: PET/CT-score; Model 2: PET/CT radiomics nomogram). DCA, decision curve analysis.

The diagnostic efficiency of each model in the training and verification cohorts is shown in **Table 6** and **Figure 5**. The sensitivity, specificity, and accuracy of conventional PET/CT diagnosis of LNM for predicting LNM were 43.8%, 88.9%, and 58.9% in the training cohort; 35.9%, 85.0%, and 52.5% in the internal validation cohort; and 42.8%, 80.0%, and 54.8% in the external validation cohort, respectively. The AUCs for predicting LNM based on the ^18^F-FDG PET/CT-score in the training cohort, internal validation cohort, and external validation cohort were 0.792 (95% CI: 0.712–0.870), 0.803 (95% CI: 0.681–0.924), and 0.762 (95% CI: 0.579–0.945), respectively. The AUCs of radiomics nomogram for predicting LNM were 0.861 (95% CI: 0.799–0.924), 0.889 (95% CI: 0.800–0.976), and 0.897 (95% CI: 0.683–0.948) in the training, internal validation, and external validation cohorts, respectively. The sensitivity, specificity, and accuracy of the radiomics nomogram for predicting LNM were 85.4%, 71.1%, and 80.6% in the training cohort; 82.1%, 85.0%, and 83.1% in the internal validation cohort; and 95.2%, 80.0%, and 90.3% in the external validation cohort, respectively. Radiomics nomogram shows significant advantage over PET/CT alone and radiomics for predicting LNM in GC, with a remarkable improvement of sensitivity and AUC in the training cohort (sensitivity: 85.4% *vs*. 74.2% *vs*. 43.8%, respectively; AUC: 0.861 *vs*. 0.792 *vs*. 0.664, respectively), internal validation cohort (sensitivity: 82.1% *vs*. 74.4% *vs*. 35.9%, respectively; AUC: 0.889 *vs*. 0.803 *vs*. 0.605, respectively), and external validation cohort (sensitivity: 95.2% *vs*. 71.4% *vs*. 42.8%, respectively; AUC: 0.897 *vs*. 0.762 *vs*. 0.614, respectively), while the specificity slightly decreased. Decision curve analysis (DCA) showed that the PET/CT radiomics nomogram had a higher net benefit than the PET/CT-score across the majority of the range of reasonable threshold probabilities in the training and validation cohorts, which indicated that the radiomics nomogram based on ^18^F-FDG PET/CT radiomics has good clinical utility (**Figure 6**).

**Table 6:** Diagnostic efficiency of each model in training and verification cohorts.

Cohorts	Models	PPV	NPV	PLR	NLR	Sensitivity (%)	Specificity (%)	Accuracy (%)	AUC (95% CI)
Training cohort	Conventional PET/CT diagnosis of LNM	88.6	44.4	3.94	0.63	43.8	88.9	58.9	0.664 (0.594–0.733)
	PET-score	91.8	48.2	5.69	0.54	50.6	91.1	64.2	0.767 (0.685–0.851)
	PET/CT-score	84.6	58.9	2.78	0.35	74.2	73.3	73.9	0.792 (0.712–0.870)^*^
	PET/CT radiomics nomogram	85.4	75.0	2.96	0.21	85.4	71.1	80.6	0.861 (0.799–0.924)^#^
Internal validation cohort	Conventional PET/CT diagnosis of LNM	82.4	40.5	2.39	0.75	35.9	85.0	52.5	0.605 (0.494–0.715)
	PET-score	92.6	56.3	6.41	0.40	64.1	90.0	72.9	0.797 (0.673–0.922)
	PET/CT-score	87.9	61.5	3.72	0.32	74.4	80.0	76.3	0.803 (0.681–0.924)^*^
	PET/CT radiomics nomogram	91.4	70.8	5.47	0.21	82.1	85.0	83.1	0.889 (0.800–0.976)^#^
External validation cohort	Conventional PET/CT diagnosis of LNM	81.8	40.0	2.14	0.71	42.8	80.0	54.8	0.614 (0.445–0.784)
	PET-score	92.3	50.0	5.71	0.48	57.1	90.0	67.7	0.779 (0.608–0.950)
	PET/CT-score	83.3	53.9	2.38	0.41	71.4	70.0	71.0	0.762 (0.579–0.945)^*^
	PET/CT radiomics nomogram	91.0	88.9	4.76	0.06	95.2	80.0	90.3	0.897 (0.683–0.948)^#^

PPV, positive predictive value; NPV, negative predictive value; PLR, positive likelihood ratio; NLR, negative likelihood ratio; AUC, area under the curve.

^*^ Compared to conventional PET/CT diagnosis of LNM, p < 0.05.

^#^ Compared to PET/CT-score, p < 0.05.

## Discussion

In the present study, we constructed a radiomics nomogram by combining ^18^F-FDG PET/CT radiomics signatures and traditional clinical risk factors (CA19-9 and conventional PET/CT diagnosis of LNM), which provides additional information for ^18^F-FDG PET/CT alone and radiomics alone for preoperatively evaluating LNM of GC, with higher AUC and sensitivity than ^18^F-FDG PET/CT alone and radiomics alone in the training cohort, the internal validation cohort, and the external validation cohort. Our results concluded that the radiomics nomogram could be used to predict LNM of GC preoperatively, making up for the deficiency of conventional ^18^F-FDG PET/CT diagnostic sensitivity, which is helpful for risk stratification, preoperative individualized assessment, and guiding treatment decisions.

^18^F-FDG PET/CT can quantitatively assess the glucose metabolism and reflect the total tumor burden, biologic aggressiveness, and proliferative activity of the primary tumor, which is always related to tumor LNM (15). The PET/CT radiomics features screened in this study included PET texture features (GLZLM-SZE) and CT routine parameter (CONVENTIONAL-HUQ3). The PET/CT-score increased with the increase in GLZLM-SZE and CONVENTIONAL-HUQ3, and compared with CONVENTIONAL-HUQ3 by CT, the GLZLM-SZE obtained by PET is more weighted. It may be due to the tumor cells ingesting a large amount of glucose, relying on glycolysis as the main source of energy metabolism, and converting glucose into lactate to generate adenosine triphosphate (ATP) to supply energy, leading to the malignant proliferation of tumor cells. This abnormal energy metabolism appears earlier than the morphological changes, and the radiomics features extracted by ^18^F-FDG PET can visualize and quantify the heterogeneity of energy metabolism sensitively, early, and specifically.

Other studies (29–31) also have shown that the combination of CT anatomical images and PET metabolic images for radiomics analysis is more effective in differential diagnosis and prognosis evaluation of diseases than PET alone, and the accuracy of predicting LNM is significantly better than that of conventional PET/CT diagnosis. Recently, a study using ^18^F-FDG PET/CT radiomics features (31) randomly assigned 185 patients with GC to the training cohort and verification cohort in a ratio of 8:2 and established BalancedBagging ensemble classifier for predicting LNM in GC. Although it has different sample sizes and methodology, the PET/CT-score for predicting LNM preoperatively in the present study is similar to that of the aforementioned study (31). In clinical practice, adequate analysis of clinical and imaging data can contribute to the correct diagnosis and management of GC, so we further combined PET/CT radiomics features with clinical risk factors to construct a radiomics nomogram to predict LNM. Previous studies have shown that radiomics nomograms can effectively predict LNM in GC patients (32, 33). However, most of them were based on postoperative parameters, such as tumor histological differentiation and vascular invasion, leading to poor clinical practicability. Our study established a radiomics nomogram for predicting LNM with PET/CT-score and two preoperative factors in GC. Incorporating CA19-9, conventional PET/CT diagnosis of LNM and PET/CT-score, the easy-to-use radiomics nomogram may be more accurate to predict LNM, improve the application of PET/CT imaging technology, and facilitate risk stratification as well as individualized treatment of GC patients. The DCA indicated that the nomogram had clinical practicability for predicting LNM in GC patients, which is conducive to the selection of perioperative treatment plan, individualized operation, and the scope of lymph node dissection.

There are some limitations of our study: firstly, the retrospective study was performed in two centers, and the sample size of the external validation group was relatively small, which may result in selective bias. Furthermore, the lesions without significant ^18^F-FDG uptake were excluded, and further studies should be performed to elucidate the function of the radiomics nomogram based on PET/CT in a subgroup of different pathological types. In the present study, 66.10%–67.74% of patients had LNM at diagnosis. These results are similar to or lower than those of previous reports (16, 18) and also similar to those of a clinical study on 1,456 cases of GC with LNM in China (72.9% of patients had LNM) (34); this reflects the real situation of Chinese patients with GC. Secondly, we focused on identifying the presence of LNM, without investigating the N-stage (N0 and N1–N3b) and the location of metastatic lymph nodes (16 stations). Thirdly, we applied the traditional radiomics method, and there may be an overfitting effect in the process of data processing.

## Conclusion

The radiomics nomogram based on the primary tumor of ^18^F-FDG PET/CT could be conveniently applied to facilitate the preoperative individualized prediction of LNM in GC, which has high external applicability and clinical practicability. Future multi-center and multi-disciplinary studies are warranted to verify the robustness of the radiomics nomogram.

## Data availability statement

The raw data supporting the conclusions of this article will be made available by the authors, without undue reservation.

## Author contributions

All authors were involved in drafting the article or revising it critically for important intellectual content, and all authors approved the final version to be published. Prof. Y-TW had full access to all of the data and takes responsibility for the accuracy of the data analysis. Study conception and design: X-QX and W-JY. Acquisition of data: XS. Analysis and interpretation of data: X-LS. All authors contributed to the article and approved the submitted version.

## Conflict of interest

The authors declare that the research was conducted in the absence of any commercial or financial relationships that could be construed as a potential conflict of interest.

## Publisher’s note

All claims expressed in this article are solely those of the authors and do not necessarily represent those of their affiliated organizations, or those of the publisher, the editors and the reviewers. Any product that may be evaluated in this article, or claim that may be made by its manufacturer, is not guaranteed or endorsed by the publisher.
